# Shaking Youngsters and Shaken Adults: Female Beetles Eavesdrop on Larval Seed Vibrations to Make Egg-Laying Decisions

**DOI:** 10.1371/journal.pone.0150034

**Published:** 2016-02-25

**Authors:** Raul Narciso C. Guedes, Jayne E. Yack

**Affiliations:** 1 Departamento de Entomologia, Universidade Federal de Viçosa, Viçosa, MG, 36570–900, Brazil; 2 Department of Biology, Carleton University, 1125 Colonel By Drive, Ottawa, ON, K1S 5B6, Canada; University of Natural Resources and Life Sciences, Vienna, AUSTRIA

## Abstract

Egg-laying decisions are critical for insects, and particularly those competing for limited resources. Sensory information used by females to mediate egg-laying decisions has been reported to be primarily chemical, but the role of vibration has received little attention. We tested the hypothesis that vibrational cues produced by feeding larvae occupying a seed influences egg-laying decisions amongst female cowpea beetles. This hypothesis is supported by three lines of evidence using two strains of the cowpea beetle (*Callosobruchus maculatus*), an Indian strain with choosy females and aggressively competing larvae and a Brazilian strain with less choosy females and larvae exhibiting an “accommodating” type of competition. First, in free-choice bioassays of seed selection, choosy Indian females selected control seeds (free of eggs, larvae, or egg-laying marker) over seeds with live larvae (free of eggs and egg-laying marker), but did not discriminate between control seeds and those with dead larvae. In contrast, less choosy Brazilian females showed no preference for seeds containing live or dead larvae over controls. Second, laser-doppler vibrometer recordings confirmed that larvae feeding inside seeds generate vibrations that are available to the female during egg-laying decisions. Third, during dichotomous choice experiments where artificial vibrations approximating those produced by feeding larvae were played back during seed selection, Indian females preferred immobile control seeds over vibrating seeds, but Brazilian females showed no preference. These results support the hypothesis that females use larval vibrations in their egg-laying decisions; whether these vibrations are passive cues exploited by the female, or active signals that ‘steer’ the behaviour of the female is unknown. We propose that vibration cues and signals could be important for host selection in insects, particularly those laying on substrates where visual or chemical cues may be unreliable. This seems to be the case with females of the cowpea beetle since visual cues are not important and chemical egg-marking does not last more than two weeks, allowing vibration cues to improve discrimination of egg-laying substrate particularly by choosy females.

## Introduction

In insects, egg-laying decisions of females can be critical for the survival and fitness of offspring, and research on such decisions has been central to understanding insect population dynamics, life-history evolution, insect-insect and insect-plant interactions, and pest management [1–4]. Egg-layers make choices based on a variety of factors, including the physical and chemical qualities of the substrate, risk of predation, and competition [1,4–6]. Information gathered by the female when assessing a substrate may involve one or more sensory modalities; the best studied being chemical, but visual and tactile modalities have also received attention [7–10]. Furthermore, both cues and signals are used by females to gauge conditions for decision-making regarding egg laying, where signals evolved to convey information from sender to receiver, while cues are inadvertent products of selection on another trait [1,4,9]. For example, chemical cues and signals encompass a range of compounds widely recognized as important host and egg-laying markers [7,9,11], while tactile signals seem also important for host selection among seed beetles [12,13]. Visual cues and signals have received less attention, but they were recognized as important for mediating egg laying by butterflies under risk of predation by ants [10]. Vibration signals and cues, while widespread throughout the sensory landscape of an insect [14,15] have received even less experimental attention as possible information sources to a host looking for an egg laying substrate.

Vibrational sensing and communication is widespread in insects, yet scientists are just beginning to appreciate the importance of this sensory modality. Substrate vibrations are widely available to insects living on plants, sand, soil, leaf litter, or fabricated materials such as beehives, termite mounds, or silk. Sources of vibrations important to insects may be abiotic (e.g. wind, rain), or biotic (e.g. signals or cues arising from conspecifics, predators, and even plants). Insects use vibrations in a multitude of contexts, including social communication between mates, rivals, or parents and offspring, finding food, avoiding predators, and monitoring the environment [14–17]. At present, there is limited experimental evidence that females use vibratory signals or cues to assess host location or suitability. Currently, evidence is restricted to examples of parasitic wasps using vibrations to locate hosts concealed within plant tissues [18–19]. Yet, information about host and substrate suitability could conceivably be garnered from many different vibrating sources, including vibration cues and signals generated by insect or plant hosts, or vibroecholocation signals produced by the egg-layer [14–17]. In this study, we test the hypothesis that female cowpea beetles use vibrations in egg laying decisions.

The cowpea seed beetle *Callosobruchus maculatus* (F.) (Coleoptera: Chrysomelidae: Bruchinae) provides an excellent opportunity to examine the role of vibrations in egg laying, since females can be highly selective of egg laying substrates [1,20–23], which is a peculiar trait, and vibration cues may be available from larvae [24]. *Callosobruchus maculatus* is a capital breeder (i.e., does not feed during its brief adult stage), and reproduction depends entirely on resources secured during the larval stages, which are contained within a single seed throughout development and subject to potentially fierce larval competition [25–27]. Larval competition for limited resources in these insects is a major driver for selection of egg-laying strategies, with increased egg loads (and thus competing larvae) decreasing the fitness gain of each egg laid [5,20,27–29]. This scenario requires the egg-laying female to estimate the quality of the substrate to reach optimal fitness gain [3,5,22,30]. Thus, females have been demonstrated to be ‘choosy’, and are able to assess the host, its suitability and egg load, and may even discriminate between self and non-self eggs on a seed [7,21,31,32].

Interestingly, there are large strain differences in female choosiness, which is associated with the strategy of larval competition [29,33–35]. The Indian strain exhibits contest competition with direct larval interference within the seed allowing the emergence of only one or a few larvae per seed with higher body mass and enhanced fitness [5,28,36,37]. Contest larval competition is associated with choosier females regarding their egg-laying behavior to minimize competition. The Brazilian strain on the other hand differs in larval competition and female choosiness, exhibiting scramble larval competition within the seed allowing the emergence of several larvae with lower body mass and reduced fitness per seed. Brazilian strain females also exhibit less choosy egg-laying behavior leading to multiple eggs laid on the same seed [27,28]. The choosiness of females in the seed beetle, in addition to the well-documented variability of larval competition behaviours between strains, enables comparative studies of the proximate mechanisms underlying different behaviours.

The proximate mechanisms used to assess the suitability of the egg-laying substrate in the cowpea beetle involves the assessment of the seed substrate qualities, in addition to its potential occupancy by con- and/or heterospecifics [23,38–40]. Multiple sensory modalities are therefore likely necessary to achieve such resolution among cowpea beetles for egg-laying decisions, and both chemical and tactile stimuli seem important [12,13,20]. Chemical stimuli have been the focus of attention and the existence of egg-marking pheromone was recognized in *Callosobruchus* species [7,11,41,42]. Such chemical markers however do not last more than a two weeks in *Callosobruchus* species [7,11], and are subject to high environmental influence and low heritability [42]. As a consequence, female selection of the egg laying substrate is likely compromised particularly when solely relying on such chemical markers. Therefore, larval feeding vibrations produced within the seed may provide important cues mediating egg-laying decisions, particularly for choosier females, such as those from the Indian strain. While vibrations have been proposed to mediate resource competition among larvae of the Indian strain [24], the role of vibration in mediating egg-laying decisions by adult females within this species has not been studied.

The purpose of this study was to test the hypothesis that egg-laying decisions by female beetles can be influenced by vibration cues. We use the Indian strain of the cowpea seed beetle, known to be selective in its egg laying decisions to test this hypothesis. Three types of experiments- dichotomous free-choice bioassays, laser vibrometry recordings, and playbacks- were conducted to test the following predictions: 1. females of the choosy Indian strain will discriminate between seeds with live or dead larvae; 2. vibration cues from live larvae developing within the seeds are available to females; 3. playbacks of vibrations corresponding to larval vibrations will deter Indian females from choosing a seed to lay eggs; 4. females of the Brazilian strain, known to be less discriminating should be less influenced by the presence of live larvae, and vibration playbacks.

## Materials and Methods

### Insect Rearing and Imaging

**Fig 1:**
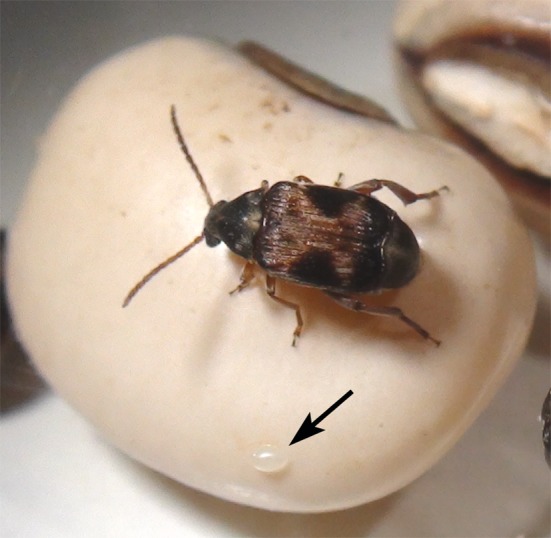
Egg laying cowpea beetle. A female *Callosobruchus maculatus* beetle inspects a seed prior to oviposition. Arrow points to a freshly laid egg on the seed surface.

Two geographic strains—Indian and Brazilian- of the cowpea beetle (*Callosobruchus maculatus*) were used in this study (Fig 1). The Indian strain was originally collected from mung beans (*Vigna radiata* (L.) Wilczek) and the related black gram (*Vigna mung* (L.) Hepper) in Tirunelveli (south India) in 1979. The Brazilian strain was originally collected from cowpeas (*Vigna unguiculata* (L.) Walpers) in Campinas (southeast Brazil) in 1975. Both were initially derived from stock cultures maintained at the UK National Resources Institute (Chatam Maritime, Kent, UK) and subsequently at the Royal Holloway School of Biological Sciences (Egham, Surrey, UK), from where they were obtained for the present study. The insect cultures of both strains were maintained in mung bean seeds (*Vigna radiata* (L.) R. Wilczek); the same procedures and densities were used in an environmentally controlled room at 12:12 h LD photoperiod at 25°C and 70 ± 10% relative humidity. These two strains were chosen based on their characteristics, which have been previously described [28–30,35,37,38,43,44]. No specific permits were required for the described studies, which were performed in the laboratory with a cosmopolitan insect pest species.

A select number of seeds that followed larval development (see vibration recordings of larvae in seeds below) were imaged within the seed using an LX-60 specimen radiography system equipped with a digital camera (Faxitron X-Ray Corp., Wheeling, IL, USA).

### Free-choice test for egg laying

**Fig 2:**
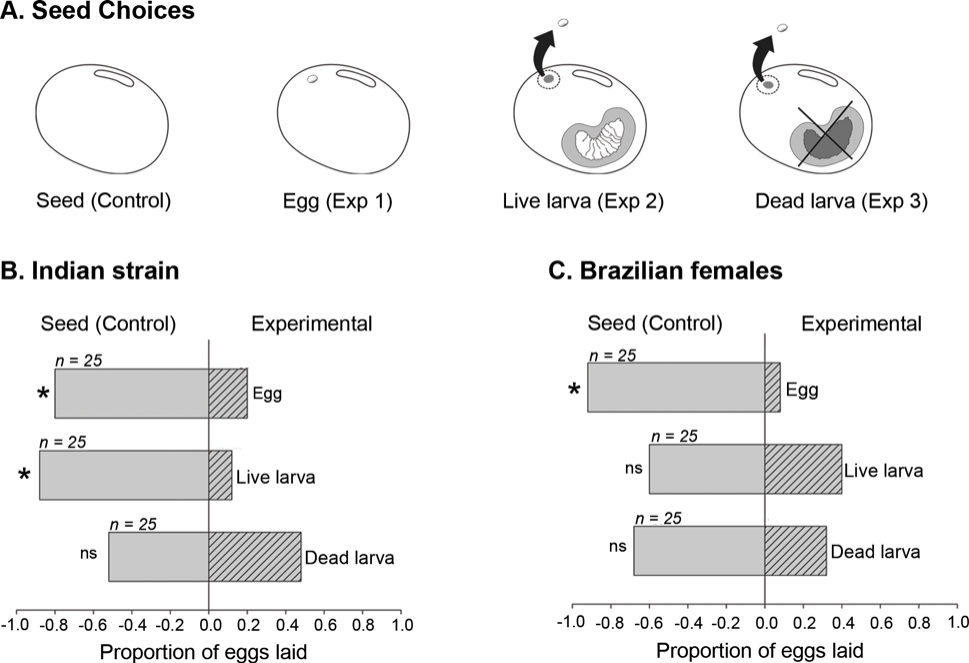
Dichotomous choice trials for *Callosobruchus maculatus* females (Indian and Brazilian strains) selecting seeds for oviposition. (A) Illustrations of the four seed conditions: Seed alone (Control), and three experimental conditions: Exp 1 seed has an egg laid by another female, Exp 2 has a living larva inside, with visual and chemical cues from the egg surface removed; Exp 3 is the same as for Exp 2 except the larva inside has been killed. (B, C) Results showing the proportion of trials where females chose the experimental condition over the control for the Indian and Brazilian strains respectively. The Indian strain avoids choosing a seed with a live larva even when external markers have been removed. Asterisks indicate significant departure from random expectation based on the adjusted *G* test (*P* < 0.05).

To test egg laying behaviour and selection characteristics of females, dichotomous free-choice tests were performed with newly mated females (48 h old) of both strains. A choice of two mung bean seeds (*Vigna radiata* (L.) R. Wilczek) placed over a filter paper (Whatman no. 1) within an open plastic Petri Dish (2.5 cm diameter) was provided for each female. The inner wall of the Petri dish was coated with Teflon PTFE (DuPont, Paulínia, SP, Brazil) to prevent the insects from escaping. Seventy-five trials were performed for each strain. In each trial, the female had the choice of laying an egg on one of two seeds: a control seed, and one of three possible experimental conditions (Fig 2A). The control was a seed free of conspecific eggs and larvae. In Experimental condition 1 (egg only), only seeds with recent (unhatched) eggs laid (< 24 hs old) were used to prevent interference from the feeding larva. In Experimental condition 2 (live larva), a female was allowed to lay eggs on the seed, but following her egg laying period, all but one of the eggs were scraped off so that just one larva would develop in the seed. After the larva hatched, its egg was also scraped off the seed leaving just the live larva within the seed. Each seed was also ‘washed’, by subjecting it to sequential 1-min immersion in ethyl acetate, methanol, and water (more apolar to polar solvent; 100 seeds per 80 mL) to remove egg-marking pheromone [7,11,41]. Seeds with a feeding larva (18–21 days old) were used in the dichotomous choice trials after confirming that larval feeding vibrations were present using a laser vibrometer (see below). In Experimental condition 3 (dead larva), the methods were identical to those in Experimental condition 2 (live larva), except that the larva inside the seed was killed at 20 days, by exposing it to -20°C for three days and checking the seed for absence of feeding vibration. For experimental conditions 1 to 3, the seed, egg and larva were always of the same strain as the female being tested.

Twenty-five different females were used for each treatment combination (three treatment conditions for each strain), resulting in a total of 150 trials, each with a different female. Each trial lasted for up to 20 min or until the first egg was laid on one of the seeds. All trials were videotaped using a camcorder (Handycam HDV 1081i/MiniDV, Sony). The video clips were subsequently imported to a computer to document time taken for each female to lay the egg (i.e., latency to egg laying) and time spent laying the egg. The fresh body mass of each egg-laying female was also recorded using an analytical balance (Sartorius CP224S, Göttingen, Germany) since mass varies with strain and may relate to fertility [26–28]. All of the trials were carried out inside an acoustic chamber (C-14A MR, Eckel, Morrisburg, ON, Canada).

### Vibration recordings of larva in seeds

To determine if larvae of both strains produce vibrations, laser vibrometry recordings were conducted on seeds containing a single developing larva over the course of the developmental period. Mung bean seeds were exposed to virgin adult females and males (24 hs old) from both strains (10 pairs per 100 seeds for 12 hs) to obtain an average of one egg per seed. If more than a single egg was laid on a seed, the excess eggs were scraped off with a scalpel before the larvae hatched (unhatched eggs remain transparent on the seed). Seeds were placed in acoustic egg-crate foam (3.81 cm thick; Foam Factory, Macomb, MI, USA) and maintained in an environmentally controlled room at 12:12 h LD photoperiod at 25°C and 70 ± 10% relative humidity. Every other day throughout the course of development (*ca*. 30 days), vibrations produced by the feeding larva within the seed were recorded with a laser vibrometer (PVD-100, Polytec Inc., Ann Arbor, MI, USA). Reflective tape (0.25 cm diameter) attached to the seed served as a laser target for the vibration recordings. The laser output was set at 20 mm/s (22 kHz low-pass filter and no high-pass filter). The signal was digitalized at 48.0 kHz and recorded to a FR-2 data recorder (FOSTEX, Norwalk, CT, USA). Temporal and spectral characteristics were analyzed using Raven Pro v. 1.3 (Cornell Laboratory of Ornithology, Ithaca, NY, USA). Power spectra were produced using 8192-point Fast Fourier Transform (Hann window; 50% overlap).

### Playback experiments

To test the effects of vibrations on seed choice, we conducted a playback experiment whereby females chose between a control seed and a seed with artificially generated vibrations. Dichotomous free-choice tests were performed with newly mated females of both strains, following the methods previously described. However, the Petri dish was modified for the playback experiments by replacing the bottom of the petri dish with foam (2 cm thick) and placing the filter paper on top. One of the two seeds resting on the filter paper was afixed by beeswax to an insect pin that was inserted through the foam and connected at its other end to a mini-shaker (Type 4810, Brüel & Kjær, Nærum, Denmark). Thus, each female had a control and an artificially vibrating seed placed 1.5 cm apart to choose as an egg laying substrate. The mini-shaker generating the seed vibration was connected to a power amplifier (Type 2718, Brüel & Kjær, Nærum, Denmark), which was driven by a waveform generator (WW 5061, Tabor Electronics, Tel Hanan, Israel). The waveform generator delivered pulse-type waves simulating the chewing events of the feeding larva (dominant frequency of 2.5 kHz at 5 events/cycle) within the mung bean seed; this was achieved by comparing the larva chewing vibration with that obtained with the waveform generator. Playback signals were monitored on an oscilloscope (TDS 2002, Tektronik, Dallas, USA). All of the trials were recorded, as described above, using a different female of each strain per trial and 25 trials (and therefore females) for each insect strain. Again each trial lasted for up to 20 min or until the first egg was laid.

### Statistical analyses

The dichotomous results of each set of trials from the free-choice experiments were subjected to the randomness *G* test using William’s procedure to correct the *G* values [45]. Time to start feeding within the seed, feeding activity during development, and latency before egg laying were subjected to time-failure analyses using Kaplan-Meier estimators and estimates of median latency and egg laying times (PROC LIFETEST) [46]. The overall similarity among curves (and median estimates) was tested by χ^2^ log-Rank test and the pairwise comparisons among curves were tested by Holm-Sidak’s test (*P* < 0.05). Correlation analysis between female body mass and latency for egg laying was also performed (PROC CORR) [46]. The dominant frequencies and bandwidths of pulses, and the durations of pulse trains in chewing vibrations produced by the feeding larvae of both strains of the cowpea beetle were subjected to multivariate analysis of variance (MANOVA), and subsequent univariate analyses of variance if significant differences were detected (PROC GLM with MANOVA statement) [46]. Male and female body mass and developmental times were subjected to two-way (univariate) analyses of variance with strain and sex as independent variables, and subsequent Tukey’s HSD test when appropriate (PROC GLM) [46]. Normality and homoscedasticity assumptions were checked (PROC UNIVARIATE) [46], but no data transformation was necessary

## Results

The results obtained support the hypothesis that female cowpea beetles of the Indian strain use vibration cues in egg laying decisions. Developing larvae generate vibrations that are detectable on the seed surface, and females choose control seeds over those containing a live larva or those that were artificially vibrated. In contrast, females of the less choosy Brazilian strain did not show evidence of using vibration cues during egg laying decisions.

### Dichotomous Choice Experiments

In dichotomous free choice trials, Indian (strain) females avoided laying eggs on seeds containing a live larva when simultaneously presented with a control seed (*G*_*adj*_ = 13.05, df = 1; *P* < 0.001; Fig 2B). However, they showed no preference between a control seed and one containing a dead larva (*G*_*adj*_ = 0.03, df = 1; *P* > 0.05; Fig 2B). Brazilian females on the other hand did not show a preference between control seeds and those containing either a live or dead larva (*G*_*adj*_ < 2.65, df = 1; *P* > 0.05; Fig 2C). Both strains avoided laying eggs on seeds containing a recently laid conspecific egg (*G*_*adj*_ > 7.71, df = 1; *P* < 0.001; Fig 2B and 2C). These results indicate that females of both strains are likely detecting the larva vibrational cues, but only Indian females are avoiding cues coming from live larvae.

**Fig 3:**
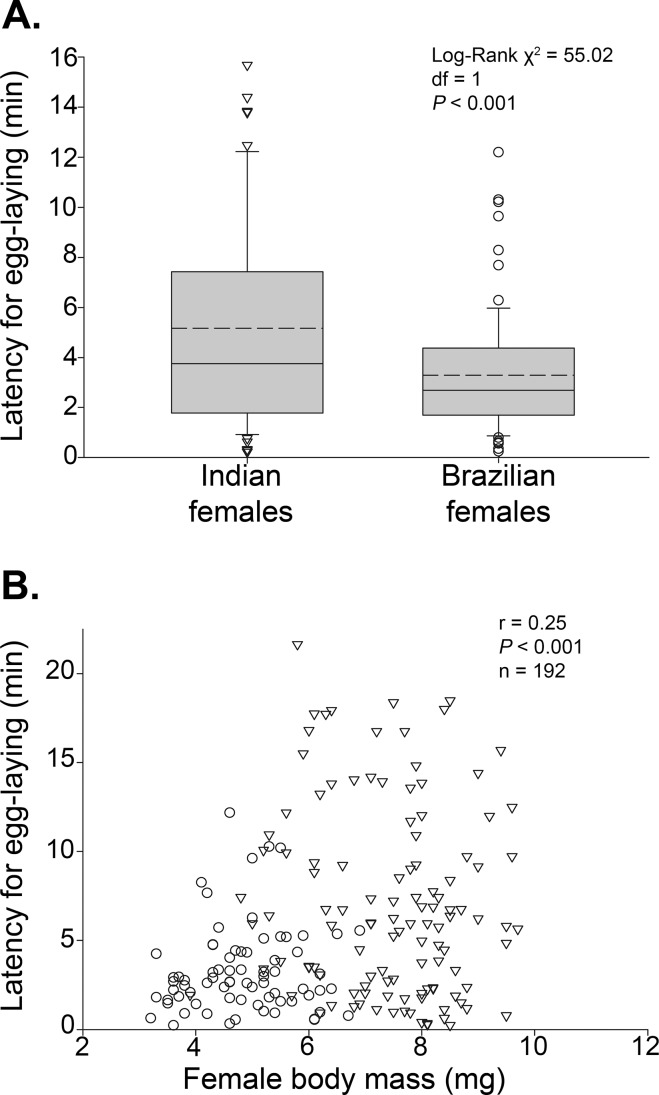
Latency to lay eggs on seeds in adult female cowpea beetles *Callosobruchus maculatus* during dichotomous choice trials. (A) A comparison of egg-laying latencies between Indian and Brazilian strain females showing that Indian females take longer to lay; the box plots indicate the median (solid line), mean (dashed line), and range of dispersion (lower and upper quartiles, and outliers) of the latency results. (B) Correlation between latency for egg laying and body mass of adult females from two strains (Indian as diamonds and Brazilian as circles).

The two strains also differed in the time it took to lay an egg (log-Rank χ^2^ = 55.02, df = 1; *P* < 0.0014) with Indian females taking significantly longer (*ca*. 40%) than the Brazilian females regardless of the egg laying choices they were facing (Fig 3A; S1 Dataset). Latency for egg laying was significantly and positively correlated with female body mass, although the correlation was weak (n = 192, r = 0.25, *P* < 0.001) (Fig 3B; S1 Dataset). This result suggests that Indian females, which are larger, are also ‘choosier’, taking longer to assess the seed prior to laying.

### Larval Vibrations

Vibrations from seeds containing a developing larva were monitored with a laser vibrometer over the course of larval development. The median time to first detect vibrations, caused by the larva feeding within the seed, did not vary with insect strain and sex (log-Rank χ^2^ = 0.77, df = 3; *P* = 0.86) exhibiting an overall median time of 4.75 days (2.5–7.0 days of 95% CI; mean of 7.55 ± 0.25 days after the larva hatched). The larvae from both strains and sexes did not differ in the length of time that they remained feeding until pupation (log-Rank χ^2^ = 1.69, df = 3; *P* = 0.64) exhibiting an overall median period of feeding of 11.90 days (8.0–16.0 days of 95% CI; mean of 19.90 ± 0.58 days after until the feeding ended).

**Fig 4:**
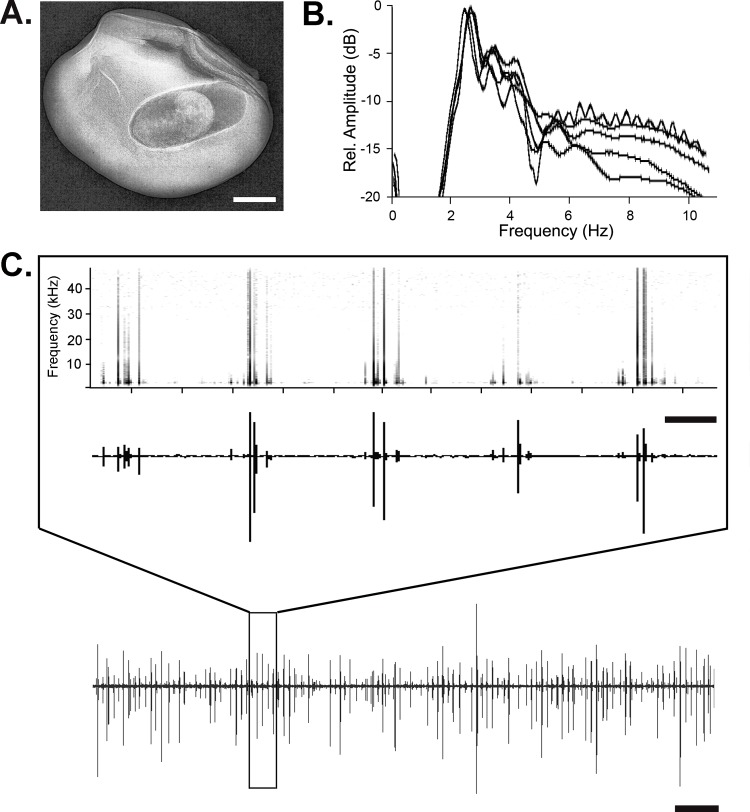
Vibrations generated by a feeding cowpea beetle larva *Callosobruchus maculatus*. (A) X-ray image of an 18-day old larva inside a mung bean seed; scale bar = 0.5 mm. (B) Power spectra of vibrations recorded from five individual larvae. (C) Bottom trace is a representative waveform recorded with a laser vibrometer from a seed containing a feeding larva (18 days old) (Scale bar: 2 sec). Top box is an expanded segment of the waveform and corresponding spectrogram showing the recurring nature of the feeding pattern and corresponding spectrogram (Scale bar: 100 ms).

Vibration characteristics were sampled from 10 individuals for each strain at 18–21 days of development. Typical vibrations, generated by an 18 day-old Indian strain larva feeding within the seed, are shown in Fig 4 (see also S1 Audio). Chewing vibrations were similar regardless of the strain and sex (Wilk’s Lambda < 0.52, F < 3.04, df_num; den_ = 4;13, *P* > 0.05) exhibiting regularly spaced pulses occurring between 4.02 ± 0.46 and 5.01 ± 0.57 events/s. Spectral analyses of the chewing vibrations indicated peak frequencies around 2.4 kHz and broad bandwidths between 0.7 and 0.9 kHz at 3 dB with relative amplitude (RMS) ranging from 8,189 to 10,150 (Table 1).

**Table 1:** Characteristics (mean ± SE) of chewing vibrations recorded from feeding larvae of the cowpea beetle *Calosobruchus maculatus*.

Sources of variation		Characteristics			
Strain	Sex	Periodicity (events/s)	Dominant frequency (Hz)	Bandwidth (Hz) at 3 dB	Amplitude (RMS)
Brazilian	Female	4.10 ± 0.54	2,400.00 ± 86.60	884.66 ± 39.44	9,354.43 ± 2,483.03
	Male	4.02 ± 0.46	2,325.00 ± 75.00	864.22 ± 37.62	10,150.22 ± 3,166.57
Indian	Female	5.01 ± 0.57	2,512.50 ± 81.01	750.34 ± 80.27	9,158.15 ± 3,079.65
	Male	4.88 ± 0.58	2,475.00 ± 91.85	700.70 ± 22.62	8,189.46 ± 727.19

The results are from 10 individual trials with larvae between 18 and 21 days old; the rates reported were sampled from the first 30 seconds of chewing.

### Playback Experiments

**Fig 5:**
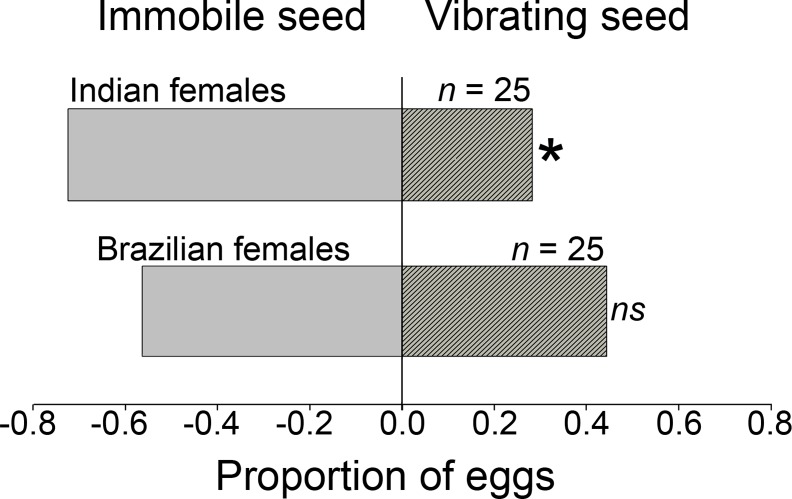
Dichotomous choice trials for *Callosobruchus maculatus* females choosing between vibrating and immobile seeds for oviposition. Proportion of females from two strains (Indian and Brazilian) selecting either immobile or artificially vibrating seeds. Asterisks indicate significant departure from random expectation based on the adjusted *G* test (*P* < 0.05).

Indian females preferred to lay their eggs on control seeds (C) that were immobile, over seeds that were artificially vibrating in playback experiments (*G*_*adj*_ = 5.15, df = 1; *P* < 0.05; Fig 5). Brazilian females on the other hand did not show a preference (*G*_*adj*_ = 2.60, df = 1; *P* > 0.05; Fig 5). The latency to lay eggs did not differ between strains subjected to the playback experiments with median latency time of 8.83 min (95% CI of 4.52–13.14 min) for the Brazilian females and 8.38 min (95% CI of 4.52–13.14 min) for the Indian females (log-Rank χ^2^ = 0.43, df = 1; *P* = 0.51). The latencies to lay eggs under the playback experiments were higher than those exhibited during regular experiments with natural vibration (or lack thereof).

## Discussion

Scientists are becoming increasingly aware that many insects use solid-borne vibrations for monitoring their environments and communicating with both con- and heterospecifics. While examples of vibratory sensing and communication in insects have been increasing over the past 20 years, there is still much to learn about which insects use vibrations, how they use them, and the sensory systems involved [14–17,47]. Here we provide experimental evidence that egg-laying decisions made by female insects can be mediated by vibratory cues.

Females of two strains of cowpea beetles—the Indian strain, and Brazilian strain- were used to test the potential that larval vibrations could mediate egg-laying decisions. Indian females have been proposed to be choosier in their selection of the egg laying substrate, as reported in previous studies [1,5,31,38]. In agreement with this, our study showed that females from the Indian strain, which exhibited higher body mass, took longer to begin laying eggs than the Brazilian females. It was predicted that the ‘choosy’ Indian strain would exhibit stronger avoidance in laying eggs in seeds containing eggs and/or larvae of the same species. Females of both strains responded similarly to seeds occupied by eggs and dead larvae, but differently to seeds occupied by live larvae. Both strains exhibited marked preference to lay eggs on control seeds (i.e., free of conspecific eggs and larva) rather than on seeds containing eggs. This result was consistent with previous findings [31,48–50], and supportive of a chemical egg laying deterrent in this species [13,51], as recognized and identified in the related species *C*. *chinenis* [11,42]. The non-discrimination of control seeds and seeds containing only a dead larva observed for both strains was also expected, as no egg laying deterrent was present due to its removal by washing the seed coat in a series of solvents, and no larval vibration was produced within the seed. Divergent responses between the beetle strains were observed; however, when the females were allowed to choose between a control seed and a seed containing a feeding larva (subjected to egg and egg laying deterrent removal)–Indian females effectively avoided seeds with a live larva, unlike Brazilian females. This finding supports the hypothesis that vibratory cues are involved in guiding egg-laying decisions in the choosier Indian strain, reinforcing that stimuli by chemical egg-laying markers fade with time, and are heavily influenced by the environment [7,42].

Vibrations recorded from seeds containing a developing larva from both strains were clearly detectable for larvae between 2 and 20 days old. Vibrations are presumed to arise from larval feeding due to the recurrence of pulses and frequency similarity to other chewing vibrations in other insects (e.g. Guedes et al. [52]). Larval vibrations were previously implicated in amelioration of resource competition among larvae of the cowpea beetle [24], although the vibrations were not characterized in that study. The vibrations produced by larval feeding within the seed in our study were characterized for males and females of both strains, but the vibration characteristics were indistinguishable among sex and strain regardless of their body mass differences. Despite the close resemblance between the larval vibrations of both strains, the choosier females of the Indian strain appeared more likely to recognize the occupancy and avoid laying eggs on larva-infested seeds. Indian females avoided laying eggs on seeds containing either eggs or larvae in contrast with Brazilian females, which did not avoid the larva-infested seeds. The hypothesis that vibrations are mediating seed choice decisions was further supported with playback experiments: Indian females avoided laying eggs on the artificially vibrating seeds, unlike Brazilian females, although the distinction was not as marked as when feeding larvae were used. Females also took longer to lay their eggs in the playback experiment than in the experiments with natural vibration (or lack thereof). These findings suggest that the artificial vibration stimulus was probably not as effective as the natural one, although robust enough to allow significant recognition by the females of the choosier Indian strain, as hypothesized. We conclude that larval chewing vibrations are important cues mediating egg-laying decisions of the choosier females of the Indian strain.

How might adult females detect vibrations? Even though there is behavioural evidence that some species of Coleoptera can detect vibrations and/or communicate using vibratory signals [14], at present, there is little direct evidence for vibration sensory organs in this insect order. In other insects, the most studied vibration receptor is the subgenual organ located in the tibia, near the ‘knee’ of the insect. To date, subgenual organs have not been reported for Coleoptera, but other leg mechanoreceptors such as the femoral chordotonal organs could be similarly used for vibration reception [17,53]. A second possibility might be the Johnston’s organ in the pedicel of the antennae, shown to function in vibration reception in some Hemiptera [54]. In the seed beetles, palpi, antennae and tarsi have all been implicated in selecting egg laying substrates [12,13], and ablation experiments indicate that maxillary and/or labial palpi play a critical role in a female’s ability to avoid egg-laden hosts [55]. Therefore, vibration mediation of egg-laying decisions may take place in any of these structures and warrants confirmation using neurophysiological and anatomical studies.

Our results provide evidence that female insects can use vibrations to make decisions on where to lay their eggs. In the Indian strain of the seed beetle whose larvae are competitively aggressive, using such vibration cues aided by egg-marking pheromone allows them to reduce egg density and thus minimize larval competition within the seed. Such observations in a cosmopolitan pest species of recognized agricultural importance also provide support for the potential use of vibration signals in pest management [56]. One possibility is the detection and monitoring of hidden insect infestation, potentiality already discerned for the cowpea beetle [57]. Another possibility is minimizing the insect infestation by reducing the likelihood of egg laying in the (artificially vibrated) seeds or delaying larva development within the seed [24]. Vibration-mediated competition may be more common than anticipated among seed beetles, which are important pest species, and this communication modality may play key roles in their ecology and potentially also in their management.

## Supporting Information

S1 Audio Larval feeding vibrations. A laser vibrometer recording of a solitary Indian strain *Callosobruchus maculatus* cowpea beetle feeding inside a seed.
(WAV)

S1 Dataset Female body mass and latency for egg-laying. Dataset for Fig 3.
(PDF)
