# Supplementary material for: Shaking Youngsters and Shaken Adults: Female Beetles Eavesdrop on Larval Seed Vibrations to Make Egg-Laying Decisions
Source: PLoS One. 2016 Feb 25;11(2):e0150034. doi: 10.1371/journal.pone.0150034 (PMC4768006; doi:10.1371/journal.pone.0150034)
Supplement: S1 Dataset — Dataset for Fig 3. (PDF) [file pone.0150034.s002.pdf]

Female body mass (mg)	Latency for egg-laying (min)
5.5	1.58
4.7	0.55
5.5	5.22
5.4	3.25
4.8	3.35
4.6	0.33
4.8	4.37
3.7	1.85
5	2.38
3.5	1.65
4.6	1.77
3.7	2.95
4.7	4.43
4.3	4.77
5.7	1.58
5.2	1.02
6.2	0.97
5.4	2.03
4.6	4.03
6.7	0.78
3.6	2.7
6.1	0.58
5.6	5.2
4.1	8.27
6.4	2.28
4.2	2.62
3.6	2.92
5.3	1.8
5.1	1.37
4.8	1.67
3.3	4.25
4.2	0.88
3.2	0.63
4.3	2.9
3.9	2.08
3.8	2.47
5.4	0.93
5.3	10.28
5.2	2.98
5.9	2.27
5.8	4.35
3.3	1.8
4.2	7.67
4.9	4.32
3.6	0.23
5.2	5.1
4.9	2.6
5	9.62
4.6	2.67
3.8	2.75
4.5	2.37
6.2	2.18
6.5	5.37
5.5	10.2
5	6.27
4.4	3.35

Latency for egg-laying (min)	
Campinas	South India
1.58	1.43
0.55	4.48
5.22	6.36
3.25	6.22
3.35	6.25
0.33	6.88
4.37	2.38
1.85	3
2.38	13.85
1.65	1.28
1.77	9.72
2.95	3.85
4.43	0.28
4.77	3.07
1.58	5.53
1.02	1.18
0.97	18
2.03	0.98
4.03	0.33
0.78	7.43
2.7	1.88
0.58	5.95
5.2	9.22
8.27	7.75
2.28	10.9
2.62	4.47
2.92	1.93
1.8	2.45
1.37	2.3
1.67	11.7
4.25	1.88
0.88	0.93
0.63	14.4
2.9	5.65
2.08	9.72
2.47	6.75
0.93	0.77
10.28	13.8
2.98	3.32
2.27	2.73
4.35	7.42
1.8	1.91
7.67	1.13
4.32	4.85
0.23	0.23
5.1	3.33
2.6	7.35
9.62	4.75
2.67	0.63
2.75	1.13
2.37	12.48
2.18	15.68
5.37	1.73
10.2	1.78

4.3	3.2
4.4	5.73
5.9	5.27
3.8	0.9
6.2	3.13
4.3	4.75
5.2	2.62
4.6	3.3
6	1.92
6.1	0.55
6.9	5.55
3.6	2.22
4.6	12.18
5.4	3.88
5.2	3.23
4.6	2.65
4.3	2.9
3.5	1.47
4	1.42
7.2	16.75
6.2	13.22
5.3	10.93
7.1	5.95
6.6	6.73
6.1	17.73
5.5	3.83
5.6	9.93
4.8	7.42
5.9	15.5
6	3.5
6	3.55
6.3	17.72
7.5	18.37
5.8	21.63
5.2	3.4
5	5.95
6.1	8.83
5.2	10.07
6	16.8
5.3	6.4
6.1	9.37
6.4	5.85
7.5	5.25
6.1	3.5
6.9	1.43
6.9	4.48
8.5	6.36
9	6.22
7.5	6.25
8.2	6.88
8.8	2.38
7.1	3
8	13.85
6.8	1.28
8.8	9.72
8.3	3.85
8.1	0.28
6.2	3.07

6.27		1.5
3.35		12.17
3.2		2.35
5.73		4.97
5.27		2.83
0.9		9.02
3.13		2.33
4.75	.	
2.62	.	
3.3	.	
1.92		2.28
0.55		0.93
5.55		12.03
2.22		3.75
12.18		1.03
3.88		5.75
3.23	.	
2.65	.	
2.9	.	
1.47	.	
1.42	.	

7.6	5.53
8.8	1.18
8.4	18
7.5	0.98
8.1	0.33
8.3	7.43
7.4	1.88
7.8	5.95
6.6	9.22
8.2	7.75
7.9	10.9
8.4	4.47
3.9	1.93
7	2.45
8.2	2.3
7.8	11.7
8.6	1.88
6.2	0.93
9	14.4
9.7	5.65
9.6	9.72
6.3	6.75
9.5	0.77
6.4	13.8
7.3	3.32
7.4	2.73
7.9	7.42
5.7	1.91
8.4	1.13
9.5	4.85
8.5	0.23
8.6	3.33
7.1	7.35
8.3	4.75
8.4	0.63
7.2	1.13
9.6	12.48
9.4	15.68
7.7	1.73
8	1.78
8.7	1.5
5.6	12.17
8.2	2.35
8	4.97
7.5	2.83
7.8	9.02
8.2	2.33
8.5	.
6.4	.
8.2	.
8.1	2.28
7.8	0.93
8	12.03
8	3.75
7.7	1.03
8.3	5.75
7.4	.
8.7	.

8	.
7.7	.
9.1	.
9.5	5.8
7.3	13.92
6.8	2.05
7.6	8.52
7	2.05
8.5	6.72
6.4	17.93
6.8	14.03
7.7	16.75
7.5	7.22
8.5	18.48
8	6.9
6.4	1.35
8	2.02
7.9	9.25
9	9.15
7.8	13.57
8	0.4
8.5	8.38
7.1	5.98
7.1	14.17
9.2	11.98
8.1	5.95
8.7	6.75
7.9	14.82
